# Interactions between epigenetics and metabolism in cancers

**DOI:** 10.3389/fonc.2012.00163

**Published:** 2012-11-15

**Authors:** Jihye Yun, Jared L. Johnson, Christin L. Hanigan, Jason W. Locasale

**Affiliations:** ^1^Department of Systems Biology, Harvard Medical SchoolBoston, MA, USA; ^2^The Sidney Kimmel Comprehensive Cancer Center, The Johns Hopkins University School of MedicineBaltimore, MD, USA; ^3^Division of Nutritional Sciences, Cornell UniversityIthaca, NY, USA

**Keywords:** Warburg effect, metabolic signaling, NAD metabolism, α-ketoglutarate and cancer, TCA cycle, histone modifications, IDH mutations

## Abstract

Cancer progression is accompanied by widespread transcriptional changes and metabolic alterations. While it is widely accepted that the origin of cancer can be traced to the mutations that accumulate over time, relatively recent evidence favors a similarly fundamental role for alterations in the epigenome during tumorigenesis. Changes in epigenetics that arise from post-translational modifications of histones and DNA are exploited by cancer cells to upregulate and/or downregulate the expression levels of oncogenes and tumor suppressors, respectively. Although the mechanisms behind these modifications, in particular how they lead to gene silencing and activation, are still being understood, most of the enzymatic machinery of epigenetics require metabolites as substrates or cofactors. As a result, their activities can be influenced by the metabolic state of the cell. The purpose of this review is to give an overview of cancer epigenetics and metabolism and provide examples of where they converge.

## Introduction

Epigenetics is defined as heritable changes in gene expression without alterations in the underlying genetic material (Morgan et al., 2005). Modifications include DNA methylation and covalent post-translational modifications of histones such as acetylation, methylation, phosphorylation, ubiquitination, phosphorylation, and crotonylation (Sharma et al., 2010; Tan et al., 2011). Since every cell in the body has the same genetic code, epigenetic regulation of gene expression plays a large role in determining cellular identity. Failure of proper maintenance of cellular epigenetic status can, thus, result in loss of tissue identity or aberrant signaling pathways that lead to developmental defects or disease states such as diabetes and cancer (Esteller, 2008; Slomko et al., 2012). It is now well accepted that cancer initiation and progression are driven by a series of genetic and epigenetic alterations that cause either activation of oncogenes or inactivation of tumor suppressor genes. Much of the recent excitement in the field of cancer epigenetics lies in the reversible nature of epigenetic alterations; unlike genomic mutations, these changes can theoretically be reversed by epigenetic therapy. Recently, four drugs that target the epigenetic machinery have been approved by the FDA for cancer treatment and have demonstrated prolonged survival and lower toxicity than conventional chemotherapy (Kelly et al., 2010; Baylin and Jones, 2011). Despite intensive research and remarkable advances in our understanding of epigenetics, the mechanisms and regulators that trigger pathological epigenetic reprogramming in cancer remains poorly understood.

Another emerging hallmark of cancer is the metabolic rewiring within cancer cells (Hanahan and Weinberg, 2011). More than 80 years ago, the biochemist Otto Warburg observed that cancer cells consume abnormally large amounts of glucose and produce high amounts of lactate even in the presence of oxygen (Warburg, 1956). Research over the past few years has reinforced this observation and furthermore revealed altered metabolism of lipids, amino acids, and nucleotides in cancer cells (Vander Heiden et al., 2009; Koppenol et al., 2011; Ward and Thompson, 2012). Interestingly, emerging evidence has suggested that epigenetics and metabolism might be tightly linked in cancer development (Teperino et al., 2010; Katada et al., 2012; Lu and Thompson, 2012). Epigenetic alterations in cancer can affect metabolic status directly by regulating the expression of metabolic enzymes, thus contributing to the metabolic reprograming in cancer (Goel et al., 2003; Chen et al., 2011; Wolf et al., 2011). Reciprocally, metabolic reprograming in cancer can affect epigenetic status which in turn alters the gene expression of oncogenes and/or tumor suppressors or chromatin structures (Hitchler and Domann, 2009; Lu and Thompson, 2012; Wellen and Thompson, 2012). In fact, virtually all epigenetic enzymes rely on metabolites as co-factors or substrates (Locasale and Cantley, 2011). In this review, we will focus on the interaction between epigenetics and metabolism. We will first discuss the interaction of epigenetics and metabolism-related to cancer development. Then, we will highlight recent work showing how alterations in cancer metabolism can shift the concentrations of co-factors or substrates and thus influence epigenetic gene regulation and chromatin structure.

## Epigenetic reprograming in cancer

### DNA methylation

In mammalian DNA, the fifth position carbon of cytosine within cytosine guanine (CpG) dinucleotides can become methylated (Esteller, 2008). There are dense regions of these CpG dinucleotides throughout the genome. These dense regions are called “CpG islands” and defined by a GC percentage and an observed-to-expected CpG ratio (Takai and Jones, 2002). CpG islands are estimated to occupy 50–70% of human gene promoters and under most conditions, methylation of CpG islands at gene promoters is associated with gene silencing (Ulrey et al., 2005). DNA methylation is catalyzed by a family of DNA methyltransferases (DNMTs), which transfers a methyl group, donated by S-adenosylmethionine (SAM), to the fifth position carbon of cytosine. There are three catalytically active DNMTs in the mammalian genome: DNMT1, DNMT3A, and DNMT3B (Bestor, 2000). DNMT1 is primarily known as a maintenance DNMT having a greater specificity for hemi-methylated DNA and is important for maintaining the DNA methylation patterns during replication. DNMT3A and 3B are considered primarily de novo methyltransferases. While the enzymes that carry out DNA methylation have been relatively well characterized, much less has been understood regarding how DNA undergoes demethylation. Two models, one involving a passive mechanism and the other an active mechanism, have been proposed to explain observations (Ooi and Bestor, 2008). After multiple rounds of DNA replication and cell division, methylation patterns can simply fail to be preserved in the genomes of the daughter cells if DNMT1 is persistently inhibited encompassing the passive demethylation process. This is believed to occur during multiple stages of development and cellular differentiation (Morgan et al., 2005). Alternatively, recent work has suggested that the physical removal of the 5′ methyl group from 5-methylcytosine (5meC) is also occurring (Kangaspeska et al., 2008; Metivier et al., 2008). More than one enzyme is thought to participate in this process, which involves a series of enzymatic steps at the heart of which lies the ten-eleven translocation (TET) proteins (Bhutani et al., 2011). The TET proteins (TETs 1, 2, and 3) utilize oxygen and α-ketoglutarate (α-KG) to catalyze multiple rounds of oxidation reactions, converting 5meC to 5-hydroxymethylcytosine (5hmC) and then to 5-formylcytosine (5fC) and finally to 5-carboxylcytosine (5caC) (Tahiliani et al., 2009; Ito et al., 2010, 2011; He et al., 2011). 5hmC, 5fC, and 5caC have all been identified as oxidative byproducts of cytosine upon exposure to hydroxyl radicals. As such, DNA repair enzymes are involved in the removal and subsequent restoration of unmodified cytosine (Wu and Zhang, 2010). These observations suggest a previously unappreciated role of DNA repair enzymes as the final step in reversing an important transcriptional regulatory phenomenon.

The cancer epigenome is marked by genome-wide hypomethylation and aberrant site-specific CpG island promoter hypermethylation (Sandoval and Esteller, 2012). While the underlying mechanisms that initiate these changes are still not clear, recent studies indicate that some changes occur in early-stage tumors and may contribute to cancer initiation (Esteller, 2008). Global DNA hypomethylation takes place at various genomic sequences such as repetitive elements, retrotransposons, introns, and gene deserts. Hypomethylation at these regions leads to increased genomic instability resulting in chromosomal rearrangement (Rodriguez et al., 2006). In addition, DNA hypomethylation can cause the activation of growth-promoting genes, such as R-RAS in gastric cancer, S-100 in colon cancer (Wilson et al., 2007) and melanoma-associated antigen (MAGE) in melanoma, and a loss of imprinting (LOI) in various tumors (Rainier et al., 1993; Sharma et al., 2010). More often studied is the aberrant promoter hypermethylation, which in contrast to hypomethylation, contributes to tumorigenesis by silencing tumor suppressors such as retinoblastoma (RB), cyclin-dependent kinase inhibitor 2A (CDKN2A, also called p16), mutL homolog-1 (MLH1), von-Hippel-Lindau tumor suppressor (VHL), and breast cancer-associated-1 (BRCA1) (Jones and Baylin, 2002, 2007). The reprogramming of epigenetics in cancer has been recently supported by the finding of somatic mutations in DNMT3A in acute myeloid leukemia (AML) (Ley et al., 2010). Furthermore, homozygous null mutations and chromosomal deletion involving the TET2 locus have been identified in various myeloid malignances, indicating that the impairment of the active DNA demethylation process might contribute to cancer development (Delhommeau et al., 2009; Ko et al., 2010).

### Histone modifications (acetylation and methylation)

Covalent modifications of lysine or arginine residues of histones are an integral part of epigenetics. The epigenetic status of histones have been demonstrated to influence transcription, DNA repair, and replication (Esteller, 2008). While there are a number of different types of post-translational modifications, we will focus on two of the most well-researched histone modifications in cancer in this review—lysine acetylation and methylation. Histone acetylation is a dynamic process that is regulated through the antagonistic activities of two large families of enzymes—the histone acetyltransferases (HATs) and the histone deacetylases (HDACs) (Shahbazian and Grunstein, 2007). The HATs and HDACs are also known as lysine acetyltranferserases (KATs) or lysine deacetylases (KDACs), respectively, since it is now appreciated that they acetylate- or deacetylate-numerous non-histone substrates (Kurdistani, 2007). HATs are grouped into three main subfamilies: GCN5-related N-acetyltransferase (GNAT), *MYST* histone acetyltransferase, and p300/CBP. While functionally distinct, each subfamily shares the common enzymatic activity of transferring the acetyl groups from acetyl-CoA to the lysine residues. Conversely, HDACs remove acetyl groups from lysine residues on histones. HDACs are divided into four groups (classes I–IV) (Zhang and Dent, 2005). Eleven of HDACs belong to class I, II, or IV and are dependent on Zn^2+^ (Haberland et al., 2009) The other seven members, known as the Sirtuins, belong to class III and require nicotinamide adenine dinucleotide (NAD^+^) as an essential cofactor. Generally, histone acetylation is associated with transcriptional activation whereas histone deacetylation is correlated with gene repression and silencing (Lane and Chabner, 2009).

Compared to lysine acetylation, lysine methylation has the additional complexity of undergoing multiple rounds of modification, generating three distinct states of lysine (monomethylated, dimethylated, and trimethylated lysine) (Varier and Timmers, 2011). Furthermore, the outcome of histone methylation can lead to transcriptional activation or repression depending on the position of the lysine that is modified (Vakoc et al., 2005; Berger, 2007; Bernstein et al., 2007). For instance, trimethylation of lysine (K) 4 on histone H3 (H3K4me3) is usually associated with transcriptional activation whereas H3K9me3 or H3K27me3 is strongly correlated with heterochromatin-mediated gene silencing. These modifications are carried out by histone methyltransferases (HMTs). HMTs constitute three classes of enzymes: SET domain lysine methyltransferases, non-SET domain lysine methyltransferases and arginine methyltransferases. Like DNMTs, all HMTs use SAM as a coenzyme to transfer methyl groups to lysine or arginine residues of substrate proteins. Lysine methyltransferases have remarkable target specificity, and they usually modify one single lysine on a single histone (Shi et al., 2004). Until recently, histone methylation was considered a terminal event (Takamura and Nomura, 1988). This view had changed with the discovery of lysine-specific demethylase 1 (LSD1) and JmjC (Jumonji C) domain demethylase (JHDM), collectively known as histone demethylases (HDM) (also known as lysine demethylase (KDMs) (Teperino et al., 2010). LSD1 is a highly conserved protein, homologous to other flavine adenine dinucleotide (FAD)-dependent oxidases, composed of two subdomains: a FAD-binding and a substrate-binding domain. LSD1 catalyzes demethylation of mono- and di-methylated H3K9 or K4, leading to context-dependent transcriptional activation or repression (Shi et al., 2004, 2005). JHDMs have a mechanism different from that of LSD1. Like the TET family discussed earlier, they belong to the oxygenase family and demethylate histones in an α-KG and Fe^2+^-dependent manner (Klose et al., 2006a,b; Tsukada et al., 2006).

As with DNA methylation, changes in histone modifications are also common in cancer (Kurdistani, 2007). One of the most prominent characteristics is global loss of acetylation of H4K16Ac (Fraga et al., 2005). Such loss of histone acetylation, which is mediated by HDACs, results in gene silencing. HDACs, such as HDAC1, HDAC2, HDAC6 and Sirtuins are often found overexpressed in various types of cancer (Halkidou et al., 2004; Song et al., 2005; Bolden et al., 2006; Saunders and Verdin, 2007) and thus have become a target for epigenetic therapy (Lane and Chabner, 2009). HATs which maintain histone acetylation levels are also altered in cancer. For example, aberrant formation of fusion proteins through chromosomal translocations of HATs such as E1A-binding protein p300 (EP300), nuclear receptor coactivator-2 (NCOA2), MYST3 [histone acetyltransferase (monocytic leukemia) 3] and MYST4 have been identified in hematological cancers (Yang, 2004). In addition to changes in histone acetylation, cancer cells also exhibit widespread changes in histone methylation patterns. Alterations in H3K9 and H3K27 methylation are correlated with aberrant gene silencing in many types of cancer (Nguyen et al., 2002; Valk-Lingbeek et al., 2004). The changes of histone methylation in cancer can be partially explained by anomalous expression or activity of HMTs and HDMs, due to chromosomal translocation, amplification, deletion, overexpression or silencing. For example, enhancer of zeste homolog 2 (EZH2), which encodes the H3K27 HMT, is overexpressed in solid tumors such as breast, skin, prostate, lung, and colon cancer (Bracken and Helin, 2009). Chromosomal translocation of myeloid/lymphoid or mixed lineage leukemia (MLL), which encodes the most thoroughly studied H3K4 HMT, leads to aberrant expression of various homeotic (hox) genes in leukemic progression (Krivtsov and Armstrong, 2007; Sharma et al., 2010). HDMs such as LSD1 and JHDM are also reported to be deregulated in various cancer types (Shi, 2007; Rodriguez-Paredes and Esteller, 2011; Varier and Timmers, 2011). The combinations of alterations of both the HMTs and HDMs in cancer are thought to contribute to changes in the epigenetic landscape of cancer.

## Metabolic reprogramming in cancer

Altered metabolism is often observed in cancer (Vander Heiden et al., 2009; Locasale and Cantley, 2011; Vander Heiden et al., 2011). This metabolic rewiring enables tumor cells to continuously survive and proliferate even in tumor microenvironments in which certain nutrients are limiting (Gatenby and Gillies, 2004). Normal cells mostly rely on mitochondrial oxidative phosphorylation to generate energy from glucose. In contrast, cancer cells prefer to metabolize glucose in a larger part by glycolysis, resulting in increased glucose consumption and lactate production even in the presence of ample oxygen (Vander Heiden et al., 2009). Otto Warburg first observed this phenomenon in the 1920s (Warburg, 1956). His observation, known as the Warburg effect or aerobic glycolysis, has become the basis of ^18^FDG-PET imaging, a common technology to detect and observe many tumors in current clinical practice (Hsu and Sabatini, 2008).

Importantly, growing evidence indicates that almost all oncogenes and tumor suppressors are closely involved in these metabolic reprogramming in cancer (Yun et al., 2009; Ward and Thompson, 2012). Furthermore, metabolic enzymes such as succinate dehydrogenase (SDH), fumarate hydratase (FH), isocitrate dehydrogenases 1/2 (IDH1/2) and phosphoglycerate dehydrogenase (PHGDH) have been reported to be genetically altered in various tumors (Baysal et al., 2000; Tomlinson et al., 2002; Parsons et al., 2008; Locasale et al., 2011; Mullen and Deberardinis, 2012). Together these findings indicate that the metabolic switches are not just byproducts of cancer development, but major contributors to it.

## Metabolic regulation of epigenetics in cancer

**Figure 1:**
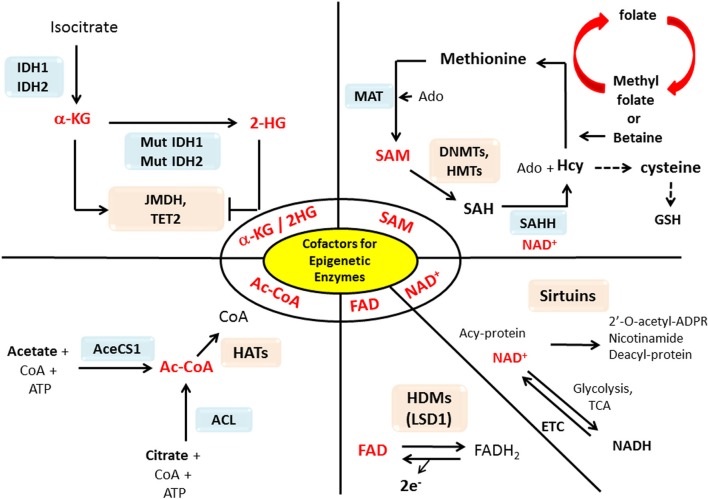
**Interactions between metabolism and epigenetics.** Metabolites that are used as substrates and cofactors for reactions that coordinate epigenetic status (red colors) are centered in this diagram, with the corresponding enzymes that utilize them shown with their chemical reactions. Abbreviation used in this figure: AceCS1, Acetyl-CoA synthetase 1; ACL, ATP-citrate lysase; Ac-CoA, Acetyl-CoA; Acy-protein, Acetylated-protein; Ado, Adenosine; α-KG, α-ketoglutarate; CoA, Co-enzyme; DNMT, DNA methyltransferase; ETC, Electron transport chain; FAD, Flavine adenine dinucleotide; FADH2, Flavin adenine dinucleotide dihydride; GSH, Glutathione; HAT, Histone acetyltransferases; Hcy, Homocystein; HDM, Histone demethylases; 2-HG, 2-hydroxyglutarate; HMT, Histon methyltransferase; IDH1/2, Isocitrate dehydrogenases 1/2; JMDH, JmjC (Jumonji C) domain demethylase; LSD1, Lysine-specific demethylase 1; MAT, Methionine adenosyltransferase; Mut, mutation; NAD^+^, Nicotinamide adenine dinucleotide; NADH, Nicotinamide adenine dinucleotide hydride; SAH, S-adenosylhomocysteine; SAHH, SAH hydrolase; SAM, S-adenosylmethionine; TCA, Tricarboxylic acid; TET2, Ten-eleven translocation 2.

The epigenetic enzymes that we discussed in the previous section require substrates or cofactors that are intermediate metabolites of cell metabolism. Theoretically, if the levels of these metabolites are elevated beyond their normal range, promiscuous activation may occur. Conversely, cancer cells may experience the depletion of metabolites needed for post-translational modifications of histones or DNA methylation (Katada et al., 2012). In this section we will discuss how the perturbation of certain metabolites in cancers may influence epigenetic reprograming in cancer in a case by case basis (summary in Table 1 and Figure 1).

**Table 1:** **Link between metabolism and Epigenetics through metabolic co-factors**.

**Metabolic co-factors**	**Enzymes that use the co-factor**	**Epigenetic function**
S-adenosyl-*L*-methionine (SAM)	DNA methyltransferase (DNMTs)	DNA methylation
	Histone methyltransferases (HMTs)	Methylation of histone or non-histone proteins
Acetyl coenzyme A (acetyl-CoA)	Histone acetyltransferases (HATs)	Acetylation of histone or non-histone proteins
Nicotinamide adenine dinucleotide (NAD^+^)	Sirtuins	Deacetylation of histone or non-histone proteins
Flavin adenine dinucelotide (FAD)	Lysine specific demthylase 1 (LSD1)	Demethylation of histone or non-histone proteins
α-Ketoglutarate (α-KG)	Ten-eleven translocation (TETs)	DNA demethylation
	JmiC histone demethylase (JHDMs)	Demehtylation of Histone or non-histone proteins

### SAM (S-adenosyl-L-methionine)

As we discussed previously, SAM is the methyl donor for reactions catalyzed by both DNMTs and HMTs. In mammalian cells, SAM is generated through the addition of ATP to methionine by methionine adenosyltransferase (MAT). In completing the methyl transfer reaction, a byproduct, S-adenosyl homocysteine (SAH) is a potent inhibitor of DNMTs and HMTs (Grillo and Colombatto, 2008). This inhibition is relieved once SAH is hydrolyzed to adenosine and homocysteine by SAH hydrolase (SAHH). Homocysteine has multiple fates (Grillo and Colombatto, 2008). It can be remethylated to restore methionine either by methionine synthase using N5-methyl tetrahydrofolate (THF) as methyl donor, or betaine homocysteine methyltransferase (BHMT) using choline-derived betaine as the methyl donor. Alternatively, homocysteine can enter the transsulfuration pathway forming cysteine, a precursor for glutathione (GSH) synthesis. These processes can be dictated in part by the given cellular requirements. Rapid cell proliferation, as seen in cancers, often results in the overproduction of reactive oxygen species (ROS) (Trachootham et al., 2009). Under these conditions, levels of GSH, a major cellular redox buffer, are elevated to minimize the deleterious effects of ROS. In this pro-oxidant state, homocysteine is diverted away from the methionine recycling pathway into the transsulfuration pathway to produce cysteine, which is a precursor and a rate limiting factor in GSH synthesis (Beutler, 1989). Conceivably, a tradeoff may be at play where there needs a decision to commit its limited resources to the collective methylation of histones or DNA or to the maintenance of its redox state. Consistent with this notion, when GSH is acutely depleted, genome-wide DNA hypomethylation follows (Lertratanangkoon et al., 1996, 1997). As discussed, cancer epigenomes are often marked by genome-wide DNA hypomethylation. Thus, some cancers may be exploiting a higher level of ROS for the purpose of reducing the production of SAM thereby dropping the activities of DNMTs and HMTs.

Recently, PHGDH, the first enzyme in the serine biosynthetic pathway, was shown to be genetically amplified in melanoma and breast cancer (Locasale et al., 2011; Possemato et al., 2011). The gene amplification was found to associate with higher levels of flux into serine and glycine metabolism. Since serine donates carbon into the folate pool, its flux can control the intracellular levels of SAM by contributing its one carbon unit to the SAM through folate pools (Mullarky et al., 2011), it is plausible that PHGDH amplification might affect the methylation status of DNA and/or histones regulating the gene expression in cancer cells.

### Acetyl-CoA (acetyl coenzyme A)

Acetyl-CoA is the acetyl-group donor for the acetylation of histones and other proteins. The intracellular acetyl-CoA levels have been observed to have more than a 10-fold variation (4–70 μM) depending on metabolic conditions and nutrient availability (Takamura and Nomura, 1988; Cai et al., 2011). Since the *K*_m_ of most HATs falls within that range, the activities of HATs are affected by fluctuations of intracellular acetyl-CoA pools. This suggests an intriguing role for acetyl-CoA as a link between cell metabolism and gene expression. Such a link was demonstrated in yeast cells where the loss of the two acetyl-CoA synthetase enzymes ACS1 and ACS2 showed dramatically reduced levels of histone acetylation and synthetic lethality resulted from deletion of the HAT, Gcn5p (Takahashi et al., 2006). Bulk histone acetylation was also shown as a response to abundant glucose that increased production of acetyl-CoA in yeast (Friis et al., 2009). In mammals, two enzymes, ATP-citrate lysase (ACL) and acetyl-CoA synthetase 1 (AceCS1), are responsible for generating the nuclear and cytosolic pools of acetyl-CoA, which can then be used for either protein acetylation or lipogenesis. Acetyl-CoA is typically generated in the tricarboxylic acid (TCA) cycle from glucose-derived pyruvate via pyruvate dehydrogenase (PDH) in the mitochondrial matrix. The excess production of acetyl-CoA in the mitochondria of proliferating cells or tumor cells results in the export of its derivative citrate to the cytosol where it is converted back to acetyl-CoA by ACL. Interestingly, Wellen et al. recently demonstrated that ACL can serve as a molecular link between cell metabolism and histone acetylation in response to growth factor stimulation (Wellen et al., 2009). Global acetylation of histones and expression of a selective subset of genes are downregulated in conditions where ACL activity is disturbed. These results from yeast and humans indicate that global and gene-specific control of transcription can be intertwined with the metabolic status of cells via acetyl-CoA. In future work, it would be interesting to investigate whether there is a causal relationship between the availability of acetyl-CoA and the global loss of acetylation of H4K16Ac which is a prevalent characteristic in many cancers (Shi et al., 2004).

### NAD^+^ (nicotinamide adenine dinucleotide)

NAD^+^ serves as a coenzyme in metabolic redox reactions, a biosynthetic precursor for several cell signaling molecules, and a cofactor for enzymes such as sirtuins (NAD^+^-dependent class III of HDACs) and poly(ADP-ribose)polymerase (Ying, 2008). The deacetylation activity of sirtuins consumes NAD^+^ as a cofactor and produces a deacetylated protein, nicotinamide, and the novel compound 2′-O-acetyl-ADP-ribose (Yang and Sauve, 2006). Since NAD^+^ is an essential cofactor for sirtuin activity, changes in levels of NAD^+^ or the NAD^+^/NADH ratio caused by metabolic status, are thought to regulate the activity of sirtuins (Lin et al., 2000; Anderson et al., 2003; Hekimi and Guarente, 2003). The link between sirtuins and metabolic status was first suggested by the findings that Sir2 in yeast is required for life span extension resulting from caloric restriction (Howitz et al., 2003; Sinclair, 2005). It is known that Sir2 activity is stimulated in nutrient deprived conditions where the ratio of NAD^+^/NADH increases. In mammals, sirtuins are key regulators of stress responses and metabolism, possibly working as a stress buffer likely through sensing changes in levels of intracellular NAD^+^ (Martinez-Pastor and Mostoslavsky, 2012). Among seven members of the mammalian sirtuin family, SIRT1 and SIRT6 are localized in the nucleus and possess deacetylase activities. The most well-studied sirtuin, SIRT1 deacetylates multiple transcription coactivators such as forkhead box, class O (FOXO), p53, peroxisome proliferator-activated receptor gamma, coactivator 1 α (PGC1-α) as well as histones (H1, H3, and H4) (Saunders and Verdin, 2007). Several sirtuins are localized to other organelles; for example, SIRT3, SIRT4, and SIRT5 are localized to mitochondria. During development, changing the NAD^+^/NADH ratio of muscle cells can alter the activity of sirtuins, thus affecting chromosome structure and gene expression (Fulco et al., 2003; Backesjo et al., 2006). The role of sirtuins in cancer is complex and multifaceted with evidence that sirtuins act as both oncogenes and tumor suppressors. For instance, some sirtuins, such as SIRT2 and SIRT6 appear to function as tumor suppressors, but others, such as SIRT1, are suggested to have a dual role (Bosch-Presegue and Vaquero, 2011). It is tempting to speculate that the increased glucose uptake and high rates of glycolysis (the Warburg effect) in cancer may influence in part sirtuins' activity by altering the ratio of NAD^+^/NADH in tumor cells. Through glycolysis, there is a net reduction NAD^+^ to NADH, thus decreasing NAD^+^/NADH ratio and leading to downregulation of the overall sirtuin activity. Reduced sirtuin activity by alterations in the NAD^+^/NADH ratio in cancer cells may result in histone hyperacetylation and decondensed chromatin structure that leads to the stimulation of gene expression (Hitchler and Domann, 2009). Further investigations are needed to understand these associations among the levels of NAD^+^, activity of sirtuins, and histone acetylation in cancer.

### FAD (flavin adenine dinucleotide)

FAD is a redox coenzyme existing in two different redox states similar to NAD^+^: oxidized form, FAD and reduced form, FADH_2_. The FADH_2_ produced in the TCA cycle, loses two electrons being oxidized back to FAD through the electron transport chain (ETC) to produce ATP by oxidative phosphorylation. In addition to its function as a redox coenzyme, FAD is an important cofactor for the demethylation of histones by the first identified HDM, LSD1 (also known as KDM1). LSD1 is a FAD-dependent monoamine oxidase which specifically removes methyl groups from mono- or dimethylated H3K4 or H3K9 through the reduction of FAD to FADH_2_ and the release of formaldehyde as a byproduct (Lu and Thompson, 2012). This can reprogram the chromatin structure and result in context-dependent activation or repression of transcription (Teperino et al., 2010). As recycling of FAD requires converting oxygen to hydrogen peroxide (H_2_O_2_), cellular redox status may affect the cellular FAD levels and thus LSD1 activity (Lu and Thompson, 2012). Oxidoreductase enzymes that use FAD as a cofactor are called flavoproteins. Interestingly, many flavoproteins are metabolic enzymes. One example is the enzyme complex SDH (complex II) that oxidizes succinate to fumarate in the TCA cycle, thereby reducing FAD to FADH_2_. Other well-known flavoproteins include acyl-CoA dehydrogenase, α-ketoglutarate dehydrogenase (α-KGDH), and a component of the PDH complex (Teperino et al., 2010). The majority of known flavoproteins are located in the mitochondria or cytosol, whereas LSD1 is one of a few flavoproteins located in the nucleus (Hino et al., 2012). Another nuclear flavoprotein is apoptosis-inducing factor (AIF) that originally localizes to the mitochondrial inner membrane and then translocate to the nucleus upon on oxidative stress or proapoptotic stimuli (Modjtahedi et al., 2006), indicating that AIF might transfer FAD pools from the mitochondria to the nucleus (Pospisilik et al., 2007; Hino et al., 2012). If so, changed activities of metabolism-related flavoproteins in the mitochondria might also influence the activity of LSD1 through competition for the cofactor, FAD suggesting the potential link between epigenetics and metabolism in cancer. Future analysis of histone demethylation by LSD1 and the cellular availability of FAD in cancer will be required to test this conceivable hypothesis.

### α-KG (α-ketoglutarate)

α-KG is a key metabolite in the TCA cycle that can be produced from glucose-derived isocitrate via an interconversion reaction catalyzed by isocitrate dehydrogenase (IDH) and it can also be produced by anaplerotic reactions such as transamination of glutamate or through glutamate dehydrogenase using glutamate as a substrate (Lu and Thompson, 2012). In addition to its roles in many metabolic pathways in the cytosol and mitochondria, α-KG can also enter the nucleus and be used as a substrate for α-KG/Fe^2+^-dependent dioxygenases such as TET and JHDM that modify epigenetic marks (Teperino et al., 2010). TET2 DNA hydroxylase converts 5meC to 5hmC at CpG dinucleotides using α-KG, oxygen and Fe^2+^ as cofactors and releases succinate and formaldehyde as byproducts. A product, 5hmC can then be an intermediate in either passive (replication-dependent) or active (TET-dependent) DNA methylation through pathways that are currently under active investigation (Tahiliani et al., 2009; He et al., 2011). α-KG is also a cofactor for another dioxygenase enzyme, JHDM that demethylate mono- di- and tri- lysine residues of histones. Recent discoveries showed that mutations in the cytosolic IDH1 and mitochondrial IDH2 resulted in a modified activity of IDH1/2 producing 2-hydroxyglutarate (2-HG) from α-KG (Dang et al., 2009; Ward et al., 2010). Since 2-HG is structurally similar to α-KG, it is reasonable to hypothesize that 2-HG can competitively inhibit α-KG-dependent enzymes such as TET and JHDM, thereby affecting epigenetic regulation.

### 2-HG (2-hydroxyglutarate)

The recent discovery of somatic mutations in the metabolic enzymes, IDH1 and IDH2 in glioblastomas has provided strong evidence for a beneficial role of altered metabolism in cancer (Balss et al., 2008; Parsons et al., 2008; Yan et al., 2009). IDH1 and IDH2 are NADP-dependent enzymes that interconvert isocitrate to α-KG in the cytosol and mitochondria, respectively, as briefly discussed in the previous section. Mutations in these enzymes have been identified in up to 80% of low-grade gliomas (Yan et al., 2009), 30% of AML (Mardis et al., 2009; Ward et al., 2010) and subsets of chondrosarcomas and lymphomas (Amary et al., 2011a; Cairns et al., 2012). Other solid tumors also acquire somatic mutations in IDH1/2 (The Cancer Genome Atlas Network, 2012). Strikingly, the mutations are single amino acid substitutions at an arginine residue in the active site of the enzyme (e.g., R132 for IDH1 and R172 in IDH2 in gliomas or R140 for IDH2 in AML). The mutations appear functionally equivalent between the IDH1 and IDH2 enzymes, and all recorded mutations were found to be heterozygous, retaining one copy of the wild-type IDH enzyme. These characteristics strongly suggested that the mutations confer a gain-of-function property to the enzyme. Indeed, recent studies have shown that mutations of IDH1 and IDH2 lead to a new enzyme activity—catalyzing the conversion of α-KG to produce 2-HG (Dang et al., 2009). In IDH1-mutated gliomas, 2-HG accumulates to concentrations of approximately 5–35 mM which is 100-fold higher than that in gliomas without these mutations (Dang et al., 2009). 2-HG was also found to accumulate to these levels in IDH1/2 mutated AML (Gross et al., 2010; Ward et al., 2010; Andersson et al., 2011) and enchondroma (Amary et al., 2011b). α-KG and 2-HG are similar in structure, and recent studies have shown that 2-HG serves as a competitive inhibitor of α-KG dependent dioxygenase enzymes. More than 60 enzymes utilize α-KG as a cofactor, and at high concentrations, 2-HG outcompetes α-KG for binding to several classes of histone demethylases such as TET2 and JHDM (Chowdhury et al., 2011). High intracellular levels of 2-HG in IDH1/2 mutant tumors are likely sufficient for potent enzymatic inhibition and suggest a possible mechanism by which IDH1/2 mutations contribute to tumorigenesis (Chowdhury et al., 2011). The biological relevance of TET inhibition by 2-HG has strong genetic evidence: gain of function mutations of IDH1/2 and loss of function of TET2 mutations were found to be mutually exclusive in a large AML cohort (Figueroa et al., 2010). Furthermore, TET2 mutant AML samples displayed an overlapping DNA hypermethylation signatures with samples having IDH1/2 mutations, and shRNA knockdown of TET2 phenocopied the effect of IDH mutant overexpression on blocking hematopoietic cell differentiation (Figueroa et al., 2010). Moreover, expression of mutant IDH in cells successfully prevented increases of 5hmC induced by TET2 (Figueroa et al., 2010). In addition, recent study showed that conditional IDH1 (R132H)-knock-in AML mice developed dysfunctional bone marrow niche and had hypermethylated histones and DNA similar to those observed in human IDH- or IDH2-mutant AML (Sasaki et al., 2012). All together, these studies strongly imply that a potential link between IDH1/2 mutations and increased DNA methylation which could be a result of the 2-HG inhibition of TET activity. In a subset of gliomas, IDH mutations are also found to be linked with DNA hypermethylation although no mutations in the TET family members have been reported so far (Noushmehr et al., 2010; Turcan et al., 2012). Interestingly, glioma samples with IDH mutations showed higher levels of H3K9me3 and H3K27me3 which might potentially explain why 2-HG can inhibit the activity of α-KG-dependent JHDMs, which demethylate histones (Lu et al., 2012). *In vitro* studies showed that 2-HG could competitively inhibit several JHDMs (Chowdhury et al., 2011; Xu et al., 2011). Further investigations of mutation studies in α-KG-dependent dioxygenase enzymes including JHDMs will be needed to genetically link IDH mutations to histone or DNA methylations in gliomas. Nonetheless, these observations regarding changes in activities of epigenetic enzymes by an oncometabolite, 2-HG continues to support direct connections between metabolic reprograming and epigenetic alterations in cancers.

## Conclusion

While we are still attempting to dissect out all of the various epigenetic mechanisms and cellular metabolic changes in cancer, it is important to understand that many of these changes and effects do not work in isolation. As discussed in this review, we are in the process of discovering the previously unappreciated link between cellular metabolism and epigenetic changes in cancer development, and we expect that many more exciting discoveries regarding these two interactions will come out in the near future. One of the challenges will be to decipher what degree and which precise regions of cancer epigenome are affected by availability of specific metabolites in each stage of cancer development and how altered metabolic flux and substrate competition affect the dynamics of specific epigenetic modifications. For therapeutic perspectives, it would be important to determine whether these epigenetic changes elicited by altered metabolites can be reversed by epigenetic therapy and whether any of the described metabolic changes have prognostic or predictive value in cancer patients. Epigenetic therapy has shown remarkable clinical activity for the treatment of various cancers (Dawson and Kouzarides, 2012). However, the biomarkers that could ultimately lead to improved patient selection and diagnosis are poorly defined. One possibility is that metabolic state of the tumor which could be assessable from primary tumor biopsies or the metabolomics of biological fluids could be used to predict the efficacy of targeted epigenetic therapies (Locasale et al., 2012). If possible, the successful application of metabolic biomarkers to predict response to epigenetic therapy would provide strong evidence for the role of metabolism in regulating epigenetics in tumors. Such biomarkers would then expand on the potential of epigenetic therapy for therapeutic gain. Although difficult, tackling these challenges will be of increasing importance as the research evolves from the understanding of these interactions into the investigation of therapeutic possibilities in cancer treatment.

### Conflict of interest statement

The authors declare that the research was conducted in the absence of any commercial or financial relationships that could be construed as a potential conflict of interest.
